# Distinct pain profiles across irritable bowel syndrome subtypes: results from the Rome Foundation Global Epidemiology Study

**DOI:** 10.1007/s11739-026-04375-1

**Published:** 2026-05-08

**Authors:** Keren Hod, Giovanni Marasco, Luigi Colecchia, Ami D. Sperber, Olafur S. Palsson, Shrikant I. Bangdiwala, Giovanni Barbara

**Affiliations:** 1 Department of Nutrition Sciences, Faculty of Health Sciences, Ariel University, Ariel, Israel; 2 Assuta Medical Centers, Tel Aviv, Israel; 3 Department of Medical and Surgical Sciences, University of Bologna, Via Massarenti, 9, 40138 Bologna, Italy; 4 IRCCS Azienda Ospedaliero-Universitaria Di Bologna, Bologna, Italy; 5 Faculty of Health Sciences, Ben-Gurion University of the Negev, Beersheba, Israel; 6 Center for Functional GI & Motility Disorders, University of North Carolina-Chapel Hill, Chapel Hill, NC USA; 7 Department of Health Research Methods, Evidence and Impact, McMaster University, Hamilton, ON Canada; 8 Population Health Research Institute, McMaster University, Hamilton, ON Canada

**Keywords:** Abdominal pain, Irritable bowel syndrome, Mediation analysis, Psychological distress, Somatization

## Abstract

Abdominal pain is the hallmark symptom of irritable bowel syndrome (IBS), yet its clinical expression varies considerably across subtypes. Despite this, few large-scale studies have compared pain features across all Rome IV-defined IBS subtypes. We aimed to delineate differences in abdominal pain patterns across IBS subtypes and evaluate the contribution of psychological comorbidities, extra-intestinal symptoms, and intermediate factors associated with psychological distress. Data were derived from the internet survey of the Rome Foundation Global Epidemiology Study, encompassing 54,127 participants from 26 countries, including 2195 IBS patients [712 constipation-predominant IBS (IBS-C), 629 diarrhea-predominant IBS (IBS-D), 712 mixed-type IBS (IBS-M), 142 unclassified-IBS (IBS-U)]. Clinical, dietary, and psychological parameters were assessed using validated questionnaires. Mediation analysis examined whether the association between IBS and psychological distress was attenuated after inclusion of illness perception, food avoidance, and work/activity impairment. IBS-C patients exhibited longer, more severe, and widespread pain, often radiating to the back and associated with greater analgesic use, while pain in IBS-D patients was linked to stool frequency and consistency changes. IBS-C patients reported greater dissatisfaction with bowel function, IBS-U patients frequently identified abdominal pain and bloating/distention as their most bothersome symptom. Psychological distress and somatic symptom burden were prevalent across all subtypes (69–78% and mean scores of 8.3–95, respectively). Mediation analyses revealed partial indirect associations of concerns about bowel function, embarrassment, and work/activity-related impairment, but not food avoidance. IBS subtypes demonstrate distinct pain and psychosocial profiles. Psychological burden is partly accounted for by illness perceptions and work/activity impairment, emphasizing importance of personalized, multimodal management strategies.

**Supplementary Information:**

The online version contains supplementary material available at https://doi.org/10.1007/s11739-026-04375-1.

## Introduction

According to the Rome IV criteria, abdominal pain occurring on average at least one day per week in the last 3 months, with symptom onset at least 6 months prior, is required for the diagnosis of irritable bowel syndrome (IBS) [1]. The pathophysiology of IBS-related abdominal pain is complex and closely intertwined with patients’ bowel habits [2]. Although abdominal pain is central to the Rome IV diagnostic framework, its clinical expression varies across IBS subtypes, suggesting that each subtype may represent a distinct pathophysiological entity [3].

The Rome Foundation Global Epidemiological Study (RFGES) estimated a worldwide IBS prevalence of 4.1%, with roughly one-third of patients classified as IBS with predominant constipation (IBS-C), one-third as IBS with mixed bowel habits (IBS-M), about one-quarter IBS with predominant diarrhea (IBS-D) and a smaller proportion (5–7%) as unclassified-IBS (IBS-U) [4]. IBS subtype status is not static, with many patients transitioning between subtypes over time, as the majority of IBS patients change their predominant bowel pattern at least once within a 1-year period, especially in the IBS-M subtype [5–7].

Beyond gastrointestinal symptoms, IBS is known to encompass a wide spectrum of extra-intestinal manifestations [8]. Psychopathological comorbidities are particularly prevalent, with estimates indicating that 40–60% of individuals with IBS experience clinically significant symptoms of anxiety or depression, at rates substantially higher than those observed in the general population [9–11]. Furthermore, among women, who constitute the majority of IBS patients, there is overlap with gynecological disorders [8, 12, 13]. Many of them report exacerbation of gastrointestinal symptoms around menses and exhibit higher rates of dysmenorrhea, premenstrual syndrome, endometriosis, vulvodynia, dyspareunia, and chronic pelvic pain. In addition, IBS frequently coexists with musculoskeletal and neurologic comorbidities. Fibromyalgia is a prominent example, as it commonly overlaps with IBS [8, 14–16]. Similarly, chronic fatigue syndrome and other somatic symptoms, including headaches, migraine, back pain, and temporomandibular joint disorder, are significantly more prevalent among patients with IBS, suggesting a possible shared pathophysiological basis involving central sensitization and autonomic dysregulation [8, 17–19].

Despite the central role of abdominal pain and its substantial clinical burden, phenotypic differences in pain characteristics across IBS subtypes remain insufficiently characterized, particularly in large, population-based cohorts using Rome IV criteria.

This study aims to address this gap by characterizing differences in abdominal pain patterns across Rome IV-defined IBS subtypes using data from a large, multinational sample. Secondary aims include assessing the prevalence of psychological comorbidities, dietary habits, and extra-intestinal conditions, as well as exploring potential intermediate factors in the relationship between IBS and psychological distress.

## Methods

### Data sources

This cross-sectional study utilized data from the internet survey arm (*n* = 54,127) of the RFGES, which was designed to assess the prevalence of 22 disorders of gut–brain interaction (DGBI) across 26 countries worldwide. Employing rigorous research methodology, the study aimed to enhance understanding of the global burden of DGBI and provide reliable regional prevalence estimates [20]. Detailed methods for this study have been described previously. In brief, data collection involved a comprehensive questionnaire comprising the 89-item Rome IV adult diagnostic questionnaire for diagnosing DGBI, as well as an 80-item supplementary questionnaire to explore associations between DGBI diagnoses and various contributing factors [20] Participants were recruited from individuals previously registered for online surveys, such as opinion polls and health studies. To minimize selection bias, the survey was presented as a general health study without specific reference to DGBI or gastrointestinal symptoms. Participants provided electronic informed consent and remained anonymous to the investigators. Ethical review was conducted independently in each country, with the study either approved or deemed exempt from ethics board oversight due to its anonymized design.

### Measurements

#### Diagnosis of IBS and assessment of abdominal pain and bowel habits

The adult Rome IV diagnostic questionnaire [21] together with a self-reported checklist of organic diseases and surgeries that could explain the gastrointestinal symptoms (e.g., celiac disease, inflammatory bowel disease, gastrointestinal malignancy, diverticulitis, or bowel resection) were used to diagnose IBS and its subtypes (IBS-C, IBS-D, IBS-M, and IBS-U). IBS subtype classification was based on current stool form and, therefore, reflects subtype status at the time of survey. Abdominal pain and bowel habits characteristics were assessed using specific items from the Rome IV modules. The overall severity of IBS symptoms was further evaluated using the IBS Severity Scoring System (IBS-SSS) [22], a validated composite measure ranging from 0 to 500, derived from five domains: pain intensity, frequency, abdominal distention, dissatisfaction with bowel habits, and interference with daily life. Dissatisfaction with bowel functioning was assessed on a 0–100 scale, with higher scores indicating greater dissatisfaction.

#### Dietary intake assessment

Weekly intake of major food groups (i.e., milk products, animal-based meats, fish, eggs, vegetables and legumes, fruits, bread, pasta, rice, and tofu) was assessed using the food frequency questionnaire (FFQ). Participants reported the number of days per week (0–7) they consumed each food group.

#### Psychological distress and extra-intestinal comorbidity

Psychological distress was evaluated using Patient Health Questionnaire-4 (PHQ-4) [23] a validated tool designed to measure symptoms of anxiety and depression over the past two weeks. The PHQ-4 consists of four questions scored on a four-point Likert scale, ranging from 0 (“not at all”) to 3 (“nearly every day”). The total score, ranging from 0 to 12, reflects levels of anxiety, depression, and overall psychological distress, with higher scores indicating greater severity of distress. We also used a dichotomous variable to distinguish between participants with any level of psychological distress (mild, moderate, or severe) and those with a normal status according to validated cut-off points [23].

Somatic symptoms were assessed using the Patient Health Questionnaire-12 (PHQ-12), a modified version of the PHQ-15 that excludes three gastrointestinal-related items [24]. This instrument covers 12 common somatic symptoms, that account for the most physical complaints encountered in primary care. The menstrual-related item was excluded from the total score to ensure comparability between men and women. In addition to calculating the total PHQ-12 score, individual symptoms were also examined, including sexual pain, fatigue, sleep disturbance, headaches, musculoskeletal pain, and menstrual cramps or problems with period (for women only).

#### Intermediate factors linking IBS diagnosis and psychological comorbidity

Work- and activity-related impairment was evaluated using the six-item Work Productivity and Activity Impairment (WPAI) questionnaire [25] which captures impairment over the previous seven days [26]. Domain scores range from 0 to 100%, with higher values reflecting greater impairment and lower productivity. Work-related items were completed only by employed respondents, while activity impairment was assessed among all participants. The WPAI module was administered only in Germany, The Netherlands, Israel, Italy, Japan, Poland, Spain, and Sweden.

Food avoidance was assessed exclusively in Italy, using the question: “In your experience, have you eliminated particular foods from your diet that you believe are responsible for your gastrointestinal disorders?”.

Illness-related cognitions or behaviors were measured using two items assessing concern and embarrassment regarding bowel functioning (i.e., “Are you concerned about your bowel functioning?”, and “Are you embarrassed to discuss your bowel functioning with others?”).

#### Statistical analyses

Descriptive analyses were described as medians with interquartile ranges (IQR) for continuous non-normally distributed variables, means ± standard deviation (SD) for continuous normally distributed variables, and frequencies with percentages for categorical variables. Data normality was assessed using the Kolmogorov–Smirnov test and by visual inspection of histograms and Q–Q plots.

Comparisons across IBS subtypes were performed using Kruskal–Wallis, one-way ANOVA, or Chi-square tests, as appropriate. When the overall test was statistically significant, post-hoc pairwise comparisons were conducted using independent-samples *t* tests, Mann–Whitney tests, or Chi-square tests, as appropriate, with Holm correction applied to adjust for multiple testing. Pairwise comparisons were not performed for outcomes with non-significant overall tests.

To explore differences in pain-related phenotypes between IBS-D and IBS-C, multiple logistic regression models were constructed, reflecting a priori comparison between these subtypes, which represent clinically and pathophysiological distinct extremes of bowel habit. IBS subtype was modeled as a binary outcome comparing IBS-C to IBS-D. Models were restricted to pain-related variables that demonstrated statistically significant differences between IBS-C and IBS-D in the initial analyses after correction for multiple testing using the Holm method, with each model including a different pain-related variable as the primary predictor of interest. Models were adjusted for covariates identified as significantly different between groups in univariate analyses (Holm-adjusted *p* < 0.005). Somatic symptom burden was additionally included as a priori covariate based on clinical relevance, irrespective of its non-significant between-group difference in univariate analyses. Variables were entered into the models using the forward method, while minimizing collinearity. Adjusted odds ratios (ORs) with 95% confidence intervals (CIs) were reported.

**Fig. 1:**
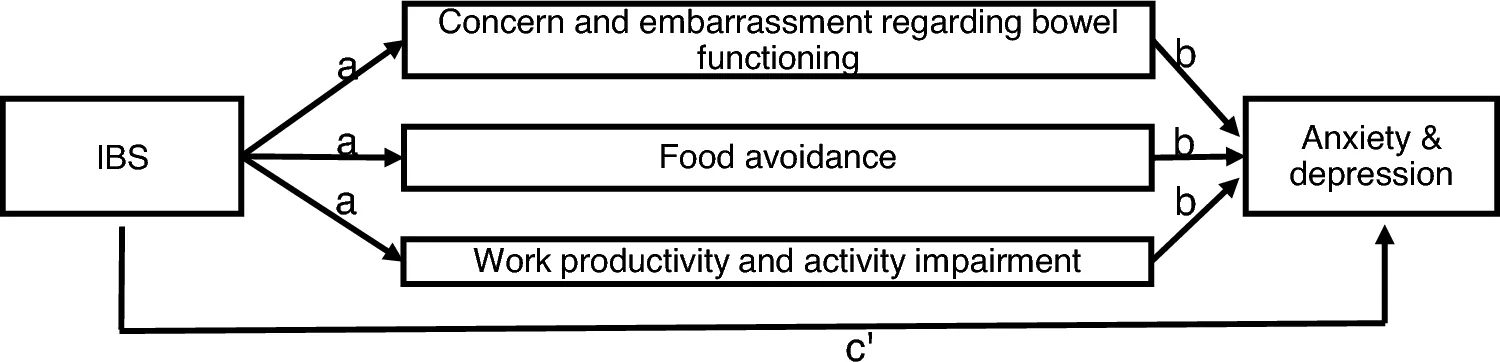
Conceptual framework of the mediation model of the current study. Anxiety and depression are associated with IBS (path c') and with intermediate factors (path b). At the same time, IBS is associated with intermediate factors (path a). When the indirect effect (a × b) is significant, while c' effect is not, we claim complete mediation, when the indirect effect (a × b), is significant as well as c' effect, we claim partial mediation

Mediation analysis examined whether the association between IBS and psychological distress was attenuated after inclusion of factors, such as work productivity, concerns and embarrassment regarding bowel functioning and food avoidance (Fig. 1). Simple mediation analysis for continuous intermediate factors was performed using the PROCESS macro in SPSS (version 4.2, Andrew F. Hayes, 2022), with bootstrapping (5000 samples). For non-parametric intermediate factors, Baron and Kenny’s method [27] was applied with three univariate regression models. Subsequently, multivariate regression model was performed to evaluate IBS and the intermediate factors as predictors of anxiety and depression. Full mediation was defined as the attenuation of the IBS-psychological distress association (path c') to non-significant after controlling for intermediate factors, while partial mediation was defined as a significant reduction in the magnitude of the association. To assess the robustness of the observed mediation patterns and to acknowledge alternative temporal orderings, additional exploratory analyses were conducted in which psychological distress was modeled as the mediating variable and each of the putative mediators was treated as the outcome. These analyses were performed using the same mediation framework.

All analyses were two-tailed (*p* < 0.05) and performed with the SPSS (Version 31, SPSS Inc., Chicago, IL).

## Results

### Demographics

**Fig. 2:**
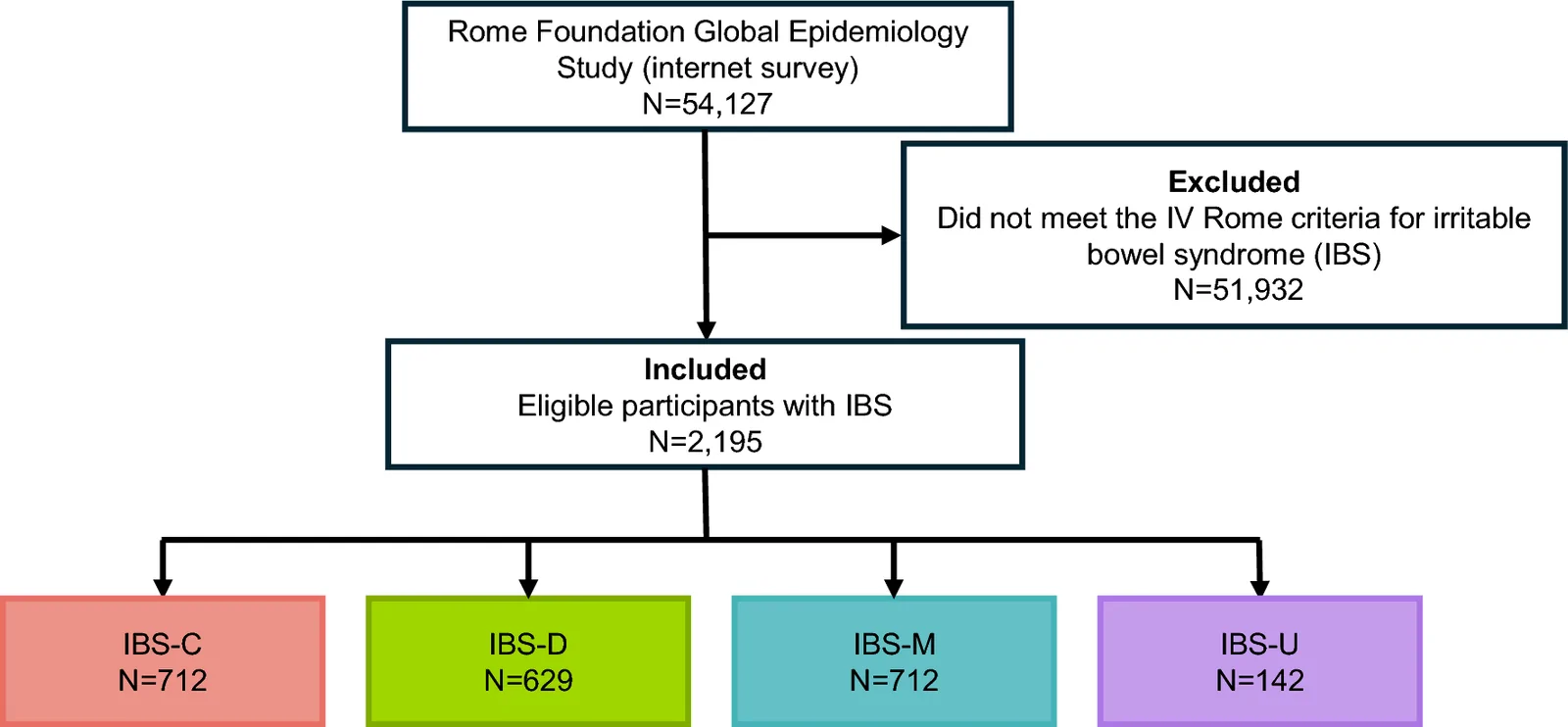
Flow diagram of the study population. The flowchart illustrates the selection of participants from the Rome Foundation Global Epidemiology Study (Internet survey, *N* = 54,127). Of these, 51,932 respondents were excluded for not meeting the Rome IV diagnostic criteria for IBS. The final analytic sample included 2195 eligible participants with IBS, categorized into four subtypes based on bowel habit: IBS-C (*n* = 712), IBS-D (*n* = 629), IBS-M (*n* = 712), and IBS-U (*n* = 142)

Of 54,127 global respondents, 712 (1.3%) met the Rome IV diagnostic criteria for IBS-C, 629 (1.2%) for IBS-D, 712 (1.3%) for IBS-M, and 142 (0.3%) for IBS-U (Fig. 2). Table 1 presents the baseline sociodemographic characteristics of the study population, with Holm-adjusted pairwise comparisons between IBS subtypes provided in Supplementary Table 1. Supplementary Table 2 summarizes dietary intake frequencies and food avoidance across IBS subtypes, with corresponding Holm-adjusted pairwise comparisons reported in Supplementary Table 3. IBS-C participants reported higher consumption of dairy (adjusted *p* = 0.002) and pasta (adjusted *p* = 0.050), with a modestly higher intake of eggs, fruits, bread, vegetables, and legumes compared to IBS-D. Food avoidance was most prevalent among individuals with IBS-C and IBS-D, reported by approximately half of participants in these groups, whereas markedly fewer individuals with IBS-M or IBS-U reported avoiding food which they believed triggered their gastrointestinal symptoms.

**Table 1:** Sociodemographic data of the study population

	IBS-C *(n* = *712)*	IBS-D *(n* = *629)*	IBS-M *(n* = *712)*	IBS-U *(n* = *142)*	Overall* p* value
Age (years), mean ± SD	38.5 ± 14.1	39.7 ± 13.5	39.5 ± 13.7	40.2 ± 14.6	0.402
Female, % (*n*)	68.4 (487)^a^	56.1 (353)^b^	65.6 (467)^a^	58.5 (83)^a,b^	< 0.001
Education (years), mean ± SD	14.4 ± 4.6	14.1 ± 4.9	14.2 ± 4.7	14.3 ± 5.1	0.726
Size of living area, % (*n*)					
City (> 50,000 inhabitants)	67.3 (479)	67.2 (423)	69.7 (496)	74.6 (106)	0.274
Town (2500–50,000 inhabitants)	23.2 (165)	24.2 (152)	23.3 (166)	20.4 (29)	0.819
Village or small town (< 2500 inhabitants)	7.7 (55)	6.7 (42)	5.5 (39)	4.9 (7)	0.310
Place in the countryside that is not a part of any city, town, or village	1.8 (13)	1.9 (12)	1.5 (11)	0.0 (0)	0.417
Current relationship status, % (*n*)					
Single	32.3 (230)	33.4 (210)	32.9 (234)	30.3 (43)	0.903
Married	45.6 (325)	45.3 (285)	41.2 (293)	50.0 (71)	0.138
Divorced	5.5 (39)	5.7 (36)	7.2 (51)	7.0 (10)	0.533
Widowed	2.2 (16)	1.7 (11)	2.5 (18)	1.4 (2)	0.712
Co-habiting (not married, but living with an adult partner)	14.3 (102)	13.8 (87)	16.3 (116)	11.3 (16)	0.357
BMI (kg/m^2^), mean ± SD	25.1 ± 5.6^a^	26.3 ± 6.4^b^	25.8 ± 6.3^a,b^	26.2 ± 5.7^a,b^	0.006
% BMI categories^c^ (*n*)					
Underweight	6.9 (43)	7.8 (43)	5.4 (33)	7.9 (10)	0.386
Normal weight	50.3 (315)^a^	39.5 (217)^b^	46.5 (284)^a,b^	39.4 (50)^a,b^	0.001
Overweight	25.6 (160)	29.1 (160)	28.8 (176)	33.9 (43)	0.213
Obesity	17.3 (108)^a^	23.6 (130)^b^	19.3 (118)^a,b^	18.9 (24)^a,b^	0.050

Pairwise comparisons were adjusted for multiple testing using the Holm method

*BMI* body mass index, *CI* confidence interval, *IBS-C* constipation predominant irritable bowel syndrome, *IBS-D* diarrhea predominant irritable bowel syndrome, *IBS-M* irritable bowel syndrome mixed type, *IBS-U* unclassified irritable bowel syndrome, *n* number

Groups sharing the same superscript letter (a, b) are not significantly different (*p* > 0.05)

^c^BMI categories: underweight: < 18.5 kg/m^2^, normal weight: 18.5–24.9 kg/m^2^, overweight: 25–29.9 kg/m^2^, and obesity: ≥ 30 kg/m^2^

### Pain characteristics

**Fig. 3:**
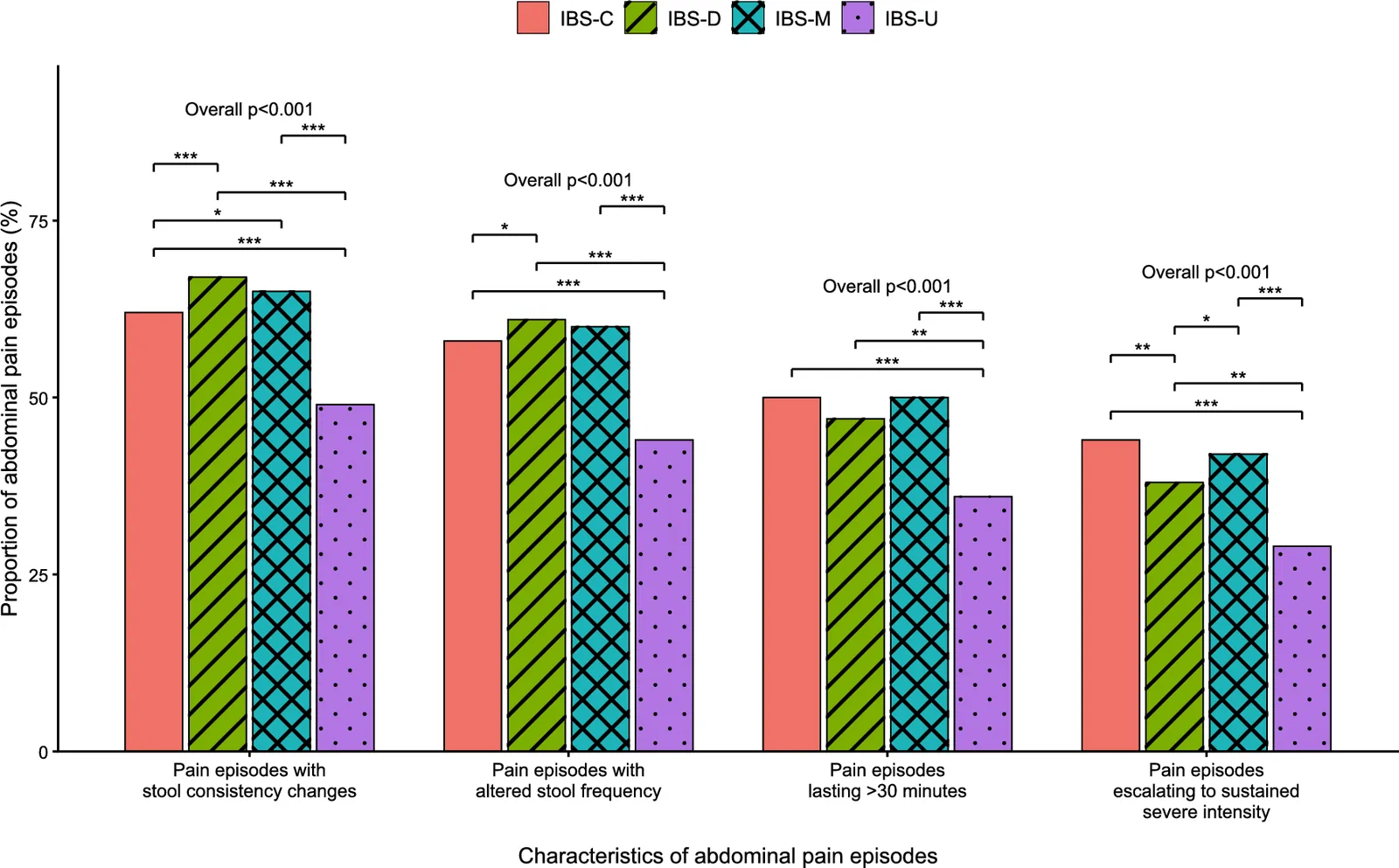
Distribution of pain-related symptoms across IBS subtypes. Percentage of abdominal pain episodes exhibiting specific features, including episodes accompanied by stool consistency changes, altered stool frequency, duration > 30 min, and escalation to sustain severe intensity. Values represent the mean participant-reported percentage (%) of abdominal pain episodes in which each feature occurred (“percent of times with pain”), calculated within each IBS subtype. For each item, the denominator reflects all abdominal pain episodes as perceived and reported by the participant. Overall differences across IBS subtypes were statistically significant for all variables (overall *p* < 0.001). Post hoc pairwise comparisons with Holm adjustment demonstrated that IBS-D and IBS-M were more frequently associated with pain occurring alongside changes in stool consistency and frequency compared with IBS-C. In contrast, IBS-C and IBS-M showed higher proportions of prolonged pain episodes (> 30 min) and episodes escalating to sustained severe intensity compared with IBS-D. IBS-U consistently exhibited the lowest proportions across all pain-related characteristics and differed significantly from the other subtypes in most comparisons. Pairwise comparisons are indicated by brackets and asterisks in the figure (*p < 0.05, **p < 0.01, ***p < 0.001; Holm-adjusted)

Pain-related characteristics varied across IBS subtypes (Fig. 3, Supplementary Tables 4, 5). The full set of corresponding Holm-adjusted pairwise comparisons is provided in Supplementary Tables 6, 7. The proportion of pain episodes escalating to a sustained severe intensity were higher in IBS-C and IBS-M compared with IBS-D (44.2%±29.4 and 42.9±29.2 versus 38.1%±29.3) with significant differences observed for IBS-C vs. IBS-D (adjusted *p*=0.003) and IBS-M vs. IBS-D (adjusted *p*=0.015). A similar pattern was observed for prolonged pain episodes (>30 min), where IBS-C and IBS-M showed higher proportions compared with IBS-U (50.7%±27.9 and 49.7%±29.3 vs. 36.3%±29.1), while no significant differences were observed between IBS-C, IBS-D, and IBS-M. Upper abdominal pain frequency was also higher in IBS-C and IBS-M (median: 4.0, IQR 2.0–5.0 for both) compared with IBS-D (median: 3.0, IQR 2.0–5.0). However, after Holm correction for multiple comparisons, only the difference between IBS-M and IBS-D remained statistically significant (adjusted *p*=0.010).

Differences also emerged in pain triggers and alleviating factors. IBS-C more often reported pain relief with postural change compared to IBS-D and IBS-M (adjusted *p*=0.012, and *p*=0.018, respectively) (Supplementary Tables 4, 6). A significantly higher proportion of pain episodes in IBS-D, relative to IBS-C, were accompanied by changes in stool consistency (adjusted *p*<0.001), and a similar pattern was observed for pain with altered stool frequency (adjusted *p*=0.023) (Fig. 3).

Patients with IBS-C reported pain radiating to the back or right shoulder region more frequently (36.7%±32.2) versus IBS-D (30.9±30.3, adjusted *p*=0.007). The higher pain burden observed in IBS-C was accompanied by greater use of analgesic medications compared with IBS-D (39.3% versus 31.8%, adjusted *p*=0.029; Supplementary Tables 4, 6).

Some overlapping pain features between IBS-C and IBS-D were also observed (Supplementary Table 5).

### Bowel habit characteristics in IBS

**Fig. 4:**
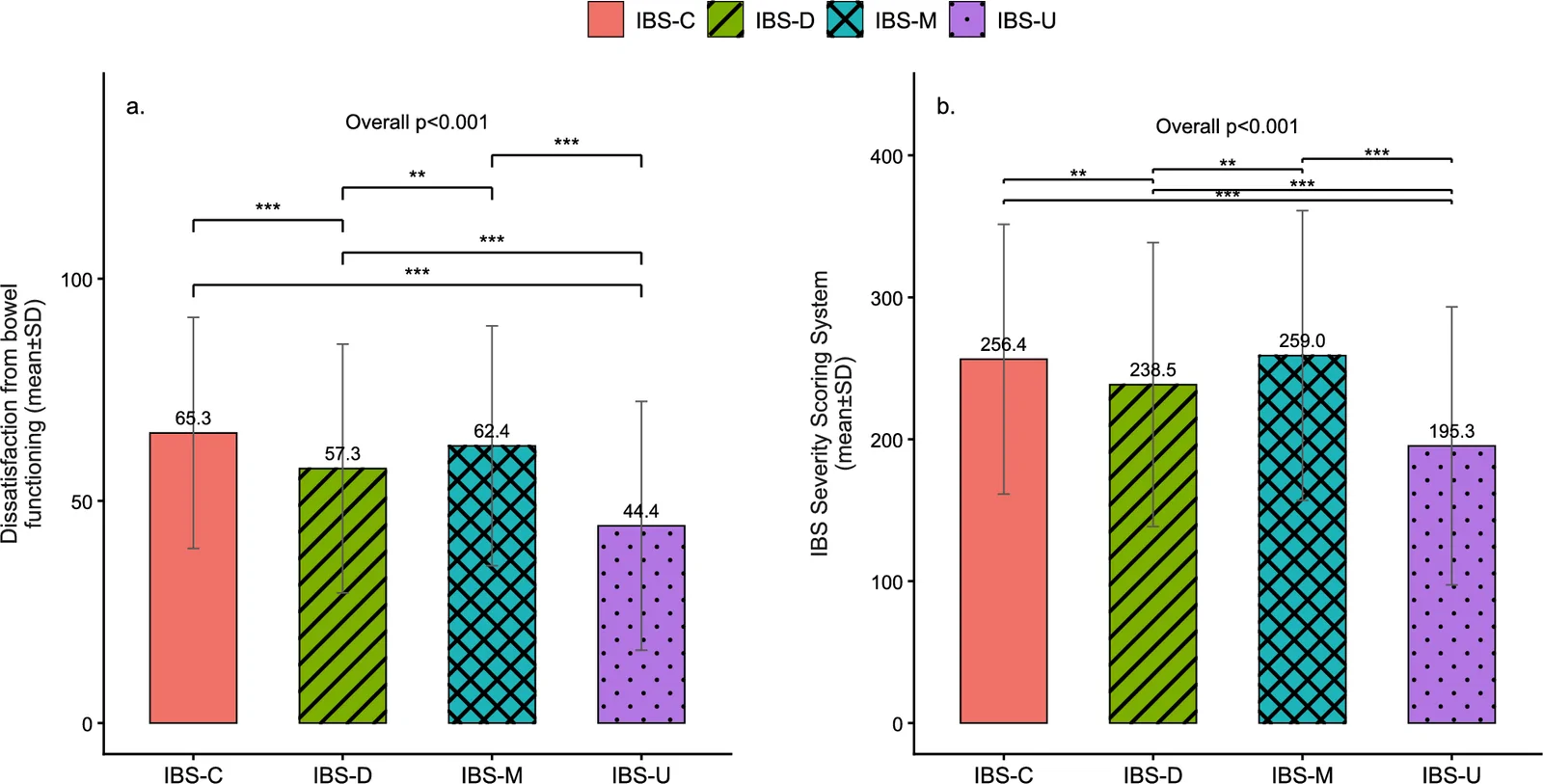
Dissatisfaction with bowel functioning and IBS severity across IBS subtypes. **a** Mean levels of dissatisfaction with bowel functioning across IBS subtypes. Overall differences were statistically significant (overall *p* < 0.001). Post hoc pairwise comparisons with Holm adjustment demonstrated that IBS-C and IBS-M reported higher dissatisfaction compared with IBS-U, and IBS-C also showed higher levels compared with IBS-D. No significant difference was observed between IBS-C and IBS-M. **b** Mean scores on the Irritable Bowel Syndrome Severity Scoring System (IBS-SSS) across IBS subtypes. Overall differences were statistically significant (overall *p* < 0.001). Post hoc analyses indicated that IBS-C and IBS-M exhibited higher severity scores compared with IBS-D and IBS-U, with IBS-U consistently showing the lowest severity. Pairwise comparisons are indicated by brackets and asterisks (*p < 0.05, **p < 0.01, ***p < 0.001; Holm-adjusted)

Patients with IBS-C reported greater dissatisfaction with bowel function (65.3±26.2) compared to IBS-D (57.3±28.4, adjusted *p*<0.001) and IBS-U (44.4±27.9, adjusted *p*=0.071) (Fig. 4a). IBS-SSS differed significantly with the highest scores in IBS-M (mean 259.0±102.9) and IBS-C (256.4±100.3), followed by IBS-D (238.5±104.2) and IBS-U (195.3±105.0) (*p*<0.001) (Fig. 4b).

Among patients with IBS-M, abdominal pain was reported as the most bothersome symptom (31.5%), a proportion nearly identical to that of bloating/distention (31.2%), indicating a similar clinical burden. The IBS-U group had the highest prevalence of abdominal pain as the leading complaint (45.1%), which was significantly higher compared with IBS-C, IBS-D, and IBS-M (adjusted *p*=0.035, *p*=0.006, and *p*=0.012, respectively). IBS-U also had the highest proportion of patients rating bloating/distention as their most bothersome symptom (34.5%), significantly higher than IBS-D and IBS-C (adjusted *p*<0.001 and *p*=0.041, respectively). In contrast, lower rates of bloating/distention as the most bothersome symptoms were observed in IBS-C (24.2%) and IBS-D (18.9%) (adjusted *p*=0.048) (Figure S1).

### Pain predictors

Multiple logistic regression models were performed to further characterize differences in pain-related features between IBS-C and IBS-D (Supplementary Table 8). Pain-related variables identified in univariate analyses (Supplementary Table 4), including sex, BMI, dairy intake, and pasta intake, were further examined in these models. Variables that reached statistical significance in univariate analyses as shown in Table 1, Supplementary Tables 2, 9 were entered as covariates. Each model examined the association of IBS subtype (IBS-C versus IBS-D) with one pain-related variable. Overall, multivariate analyses showed partial consistency with univariate findings, with several associations attenuated after adjustment. IBS-C remained independently associated with prolonged and severe pain episodes and with pain radiating to the back or to the right shoulder region. In contrast, the associations with pain relief by postural changes and with frequent use of physician-prescribed pain medication were no longer statistically significant after adjustment. IBS-D remained independently associated with pain occurring alongside changes in stool frequency and consistency.

### Psychological distress and extra-intestinal comorbidity

Psychological distress was highly prevalent across all IBS subtypes (69.0–77.9%, N.S.) (Supplementary Table 9). Depression and anxiety scores differed across IBS subtypes (overall *p*=0.001; Figure S2a), with the highest scores observed among patients with IBS-M (5.8±3.4), and lowest among those with IBS-U (4.8±3.5). Pairwise comparisons showed that IBS-M had significantly higher scores than IBS-U (adjusted *p*=0.003) and IBS-D (adjusted *p*=0.029), while IBS-C also had higher scores than IBS-U (adjusted *p*=0.029).

Somatic symptoms severity also differed across IBS subtypes (Figure S2a, Supplementary Table 9) with higher scores observed in IBS-C and IBS-M compared with IBS-U (adjusted *p*=0.035 and *p*=0.018, respectively). Among individual somatic symptoms, IBS-M showed higher rates of sleep impairment compared with IBS-U (adjusted *p*=0.033), and higher prevalence of musculoskeletal pain compared with IBS-C (adjusted *p*=0.006). Pain or problems during sexual intercourse were more frequently reported in IBS-C compared with IBS-D and IBS-U (33.3% vs. 25.0% and 20.4%, adjusted *p*=0.006 and *p*=0.017, respectively) (Supplementary Table 10).

Concerns related to bowel functioning differed across IBS subtypes (Supplementary Table 9). While high levels of concern were reported across IBS-C, IBS-D, and IBS-M (90.7%, 89.0%, and 91.6%, respectively), significantly lower rates were observed in IBS-U (69.0%), with no significant differences between IBS-C, IBS-D, and IBS-M (adjusted *p*<0.001 for all comparisons vs. IBS-U).

A similar pattern was observed for embarrassment discussing bowel function, with IBS-C and IBS-M reporting higher rates than IBS-U (60.5% and 61.4% vs. 47.2%; adjusted *p*=0.022 and *p*=0.014, respectively), while differences between IBS-C, IBS-D, and IBS-M were not statistically significant.

### Intermediate factors are associated with psychological distress in IBS: mediation analyses

Mediation analyses tested whether illness-related cognitions or behaviors showed evidence of statistical mediation of the association between IBS and psychological distress. Simple mediation models were conducted for three non-parametric intermediate factors: (1) patients’ concerns about bowel functioning, (2) embarrassment when discussing bowel habits, and (3) food avoidance due to gastrointestinal symptoms (Table 2). Across all models, the total effect of IBS on the psychological distress was significant; IBS was associated with higher odds of moderate-to-severe anxiety and depression compared with non-IBS controls (baseline OR = 4.67, *p* < 0.001). When adjusting for concerns about bowel functioning or embarrassment, the relationship between IBS and psychological distress remained significant, but was attenuated (e.g., OR reduced from 4.67 to 3.12 for concern, and to 4.16 for embarrassment, both *p* < 0.001). In contrast, food avoidance became non-significant in the adjusted model.

**Table 2:** Simple mediation analysis for the relationship between IBS and anxiety and depression

Intermediate factors (M)	1st step: OR of IBS on M (a)	2nd step: OR of M on depression and anxiety (b)	3rd step: OR of IBS on depression and anxiety (c')	4th step: ORs of the adjusted model (b, c')	Interpretation
Illness-related cognitions or behaviors					
Concerns about bowel functioning^a^	10.260***	2.892***	4.669***	2.704***, 3.124***	Partial statistical mediation
Embarrassment discussing bowel function^b^	2.475***	2.083***	4.669***	2.009***, 4.160***	Partial statistical mediation
Food avoidance^c^	2.935***	1.242*	4.669***	1.151, 6.111***	No mediation effect
Work and activity impairment^d^					
Work time missed due to health problem	6.305***	0.022***	2.513***	0.136*, 2.650***	Partial statistical mediation
Impairment while working due to health problems	19.086***	0.063***	1.469***	1.195*, 2.665***	Partial statistical mediation
Overall work impairment due to health	24.062***	0.038***	2.319***	0.913*, 3.232***	Partial statistical mediation
Activity impairment due to health problems	24.327***	0.054***	1.645***	1.312*, 2.957***	Partial statistical mediation

All analyses conducted using the Baron and Kenny’s method with bootstrapping techniques with 95% CI based on 5000 samples

*a* path a, *b* path b, *c'* path c', *M* intermediate factor, *OR* Odds ratio

^*^p < 0.05, ^**^p < 0.01, ^***^p < 0.001

^a^Concerned about bowel functioning was reported by the following question: “Are you concerned about your bowel functioning?”

^b^Embarrassed to discuss bowel functioning with others was reported by the following question: “Are you embarrassed to discuss your bowel functioning with others?”

^c^Food avoidance was reported only by the Italian participants (*n* = 2063) using the following question: “In your experience, have you eliminated particular foods from your diet that you believe are responsible for your gastrointestinal disorders?”

^d^Work productivity and impairment were assessed using the Work Productivity and Impairment (WPAI) questionnaire

We also evaluated whether work-related functioning could account for part of the observed association with psychological distress in IBS. Four parametric intermediate WPAI domains were examined: work time missed, impairment while working, overall work productivity loss, and activity impairment (Table 2). In all four models, adjustments resulted in a partial reduction in the IBS-distress association, indicating that work- and activity-related impairments were associated with attenuation of the IBS–psychological distress association.

In exploratory analyses examining alternative model specifications, in which psychological distress was modeled as the mediating variable and the putative mediators as outcomes, similar patterns of significant indirect associations were observed across most models, with the exception of food avoidance, which was not significant.

## Discussion

The present study aimed to differentiate the clinical presentation of IBS subtypes, and evaluate psychological comorbidity, extra-intestinal manifestations, and potential intermediate factors linking IBS to anxiety and depression. Overall, our findings demonstrate clear differences in abdominal pain characteristics across IBS subtypes, with IBS-C exhibiting more intense and persistent pain, and IBS-D showing pain more closely linked to bowel movements*.* A prior cross-sectional study involving 247 patients reported distinct symptom profiles across IBS subtypes [28]. However, this study was based on a relatively small sample and earlier diagnostic criteria. In line with these observations, our findings support previous studies highlighting subtype-specific pain profiles and suggest that IBS-C and IBS-D are characterized by distinct clinical patterns that may warrant tailored management approaches [28–31]. Our results also reaffirm earlier reports showing that pain in IBS-C is often more diffuse and disruptive to daily activities [32], although some studies have noted comparable pain severity across subtypes.

Despite their overall lower pain scores, IBS-U patients paradoxically identified abdominal pain as their most bothersome symptom. This likely reflects symptoms of salience rather than severity, in the absence of prominent bowel habit abnormalities, pain may become the dominant concern. This distinction underscores the multidimensional nature of symptom perception and reinforces the value of integrating both quantitative and qualitative assessments when evaluating patient burden.

Psychological comorbidities were highly prevalent across all subtypes. Anxiety and depression scores were comparable between IBS-C and IBS-D, consistent with previous work [33]. In contrast, somatic symptom burden differed modestly across subtypes, with IBS-C and IBS-M showing higher levels compared with IBS-U, suggesting a broader extra-intestinal burden in these groups. IBS-M also demonstrated higher rates of sleep disturbances and musculoskeletal pain, alongside greater illness-related concerns and embarrassment. These findings extend the existing literature showing that IBS is frequently accompanied by multisystem manifestations [17, 34, 35], supporting the importance of an integrated biopsychosocial management approach.

The complex, multifactorial pathophysiology of IBS [36–38] likely contributes to the heterogeneity of symptom profiles. Visceral hypersensitivity, more pronounced in IBS-D, may explain the stronger association between pain and bowel habit changes in this group [39], whereas prolonged transit time and increased luminal distention in IBS-C may underlie its characteristic pain pattern [39].

The gut–brain axis is central to understanding the link between IBS and psychological distress. Dysregulation of this bidirectional system can amplify both gastrointestinal and psychological symptoms [35, 40]. Our mediation analyses are consistent with this conceptual framework: work-related impairments, concerns about bowel function, and embarrassment, each demonstrated partial statistical mediation of the association between IBS and psychological distress. These findings highlight the broader psychological burden of IBS and the need to assess functional and emotional domains alongside gastrointestinal symptoms. Food avoidance did not mediate this relationship, possibly due to limited data available only in the Italian subsample. Alternative model specifications yielded similar patterns of indirect associations, supporting the possibility that these relationships reflect interconnected and potentially bidirectional processes statistically associated, rather than indicating unidirectional associations or precise causal directionality. This is consistent with the conceptualization of IBS as a DGBI, in which psychological distress may both result from and contribute to symptom perception, illness-related concerns, and functional impairment.

Our results are consistent with prior evidence that disease burden is only partially explained by gastrointestinal symptoms, and that extrinsic factors play essential roles in shaping overall quality of life [17]. These observations underscore the importance of early identification and targeted psychological interventions to improve both bowel-related and mental health outcomes.

This study has notable strengths, including a large multinational sample, standardized diagnostic tools, and comprehensive assessment of psychological and somatic variables. The mediation analyses provided insights into the associations linking IBS to psychological distress. The findings provide clinically relevant insights in a large multinational sample.

However, several limitations should be acknowledged. IBS classification was based on the Rome IV diagnostic questionnaire and self-reported exclusion of organic diseases, without clinical confirmation by a healthcare professional. Although Rome IV criteria represent the current standard for IBS diagnosis, some degree of misclassification cannot be excluded. Participants were recruited through Internet-based survey panels, which may introduce selection and coverage biases and limit representativeness within individual countries. However, evidence from the RFGES suggests that Internet-based surveys may reduce underreporting of sensitive gastrointestinal symptoms compared with face-to-face interviews, while preserving consistent patterns of disease distribution [4]. The IBS-U subgroup was relatively small, which may limit statistical power for comparisons involving this group. In addition, IBS subtype classification is based on current stool patterns, thus reflecting symptom patterns at the time of the survey rather than stable biological entities. Moreover, the cross-sectional design precludes causal inferences regarding the temporal relationships between IBS, intermediate factors, and psychological distress. These factors may have developed concurrently, and reverse causality is plausible, whereby psychological distress may influence symptom perception, illness-related concerns, or functional impairment. Future longitudinal studies are needed to clarify the causal ordering of these associations. In addition, self-reported data may introduce recall bias and affect the accuracy of symptoms and comorbidity reporting. Finally, the sample size for the mediation analysis involving dietary avoidance was limited to the Italian subsample. Had such data been available across additional countries, dietary avoidance may have emerged as a more robust intermediate factor.

In conclusion, IBS subtypes exhibit distinct pain patterns and psychological profiles, emphasizing the importance of personalized, multimodal management strategies. Identifying intermediate factors that contribute to psychological distress may guide more effective interventions. Future research should focus on evaluating the role of psychological therapies early in the disease course for IBS, while assessing both gastrointestinal and mental health outcomes.

## Supplementary Information

Below is the link to the electronic supplementary material.

Fig. S1 Most bothersome gastrointestinal symptoms across IBS subtypes. Proportion of patients with IBS identifying each symptom as the most bothersome, including abdominal pain, loose stools or increased bowel movement frequency, hard stools or absence of bowel movements for multiple days, bloating/distention, and other symptoms. Overall differences across IBS subtypes were statistically significant for all symptoms except “other symptoms” (overall p = 0.145). Post hoc pairwise comparisons with Holm adjustment demonstrated distinct subtype-specific patterns. Abdominal pain was more frequently reported as the leading complaint in IBS-U compared with IBS-D and IBS-M. Loose stools or increased bowel movement frequency predominated in IBS-D compared with all other subtypes. Hard stools were most frequently reported in IBS-C compared with IBS-D, IBS-M, and IBS-U. Bloating/distention was more commonly reported in IBS-M and IBS-U compared with IBS-D, with IBS-U also showing higher rates compared with IBS-C. Pairwise comparisons are indicated by brackets and asterisks (*p < 0.05, **p < 0.01, ***p < 0.001; Holm-adjusted). Supplementary file1 (PDF 40 KB)

Fig. S2 Psychological distress and somatic symptom burden across IBS subtypes. (a) Mean scores of anxiety and depression (left) and somatic symptom severity (right) across IBS subtypes. Overall differences were statistically significant for both measures (overall p = 0.001 and p = 0.004, respectively). Post hoc pairwise comparisons with Holm adjustment indicated modest differences in anxiety and depression scores, with higher scores observed in IBS-M compared with IBS-D and IBS-U, and lower scores in IBS-U compared with IBS-C. For somatic symptom severity, higher scores were observed in IBS-C and IBS-M compared with IBS-U, while no significant differences were found between IBS-C, IBS-D, and IBS-M. (b) Prevalence of specific somatic symptoms across IBS subtypes. Overall differences were observed for selected symptoms, including pain or problems during sexual intercourse (overall p < 0.001), trouble sleeping (overall p = 0.014), musculoskeletal pain (overall p = 0.008), and menstrual-related symptoms (overall p = 0.027), whereas no significant differences were found for fibromyalgia, fatigue, or headaches. Post hoc analyses showed that pain or problems during sexual intercourse were more frequently reported in IBS-C compared with IBS-D and IBS-U. Trouble sleeping and musculoskeletal pain were more prevalent in IBS-M compared with IBS-U and IBS-C, respectively. Pairwise comparisons are indicated by brackets and asterisks (*p < 0.05, **p < 0.01, ***p < 0.001; Holm-adjusted). Supplementary file2 (PDF 69 KB)

Supplementary file3 (DOCX 97 KB)

## Data Availability

For more information about the study database (Rome Foundation Global Epidemiology Study), we refer to the website of the Rome Foundation: https://theromefoundation.org/research-institute-rome-foundation/rome-foundation-globalepidemiology-study/. Access to the dataset is available according to the procedures outlined by the Rome Foundation Global Epidemiology Study group, upon approval of a submitted project proposal.
