# Supplementary material for: Distinct pain profiles across irritable bowel syndrome subtypes: results from the Rome Foundation Global Epidemiology Study
Source: Intern Emerg Med. 2026 May 8;21(6):2065–75. doi: 10.1007/s11739-026-04375-1 (PMC13582041; doi:10.1007/s11739-026-04375-1)
Supplement: Supplementary file 1 [file 11739_2026_4375_MOESM1_ESM.pdf]

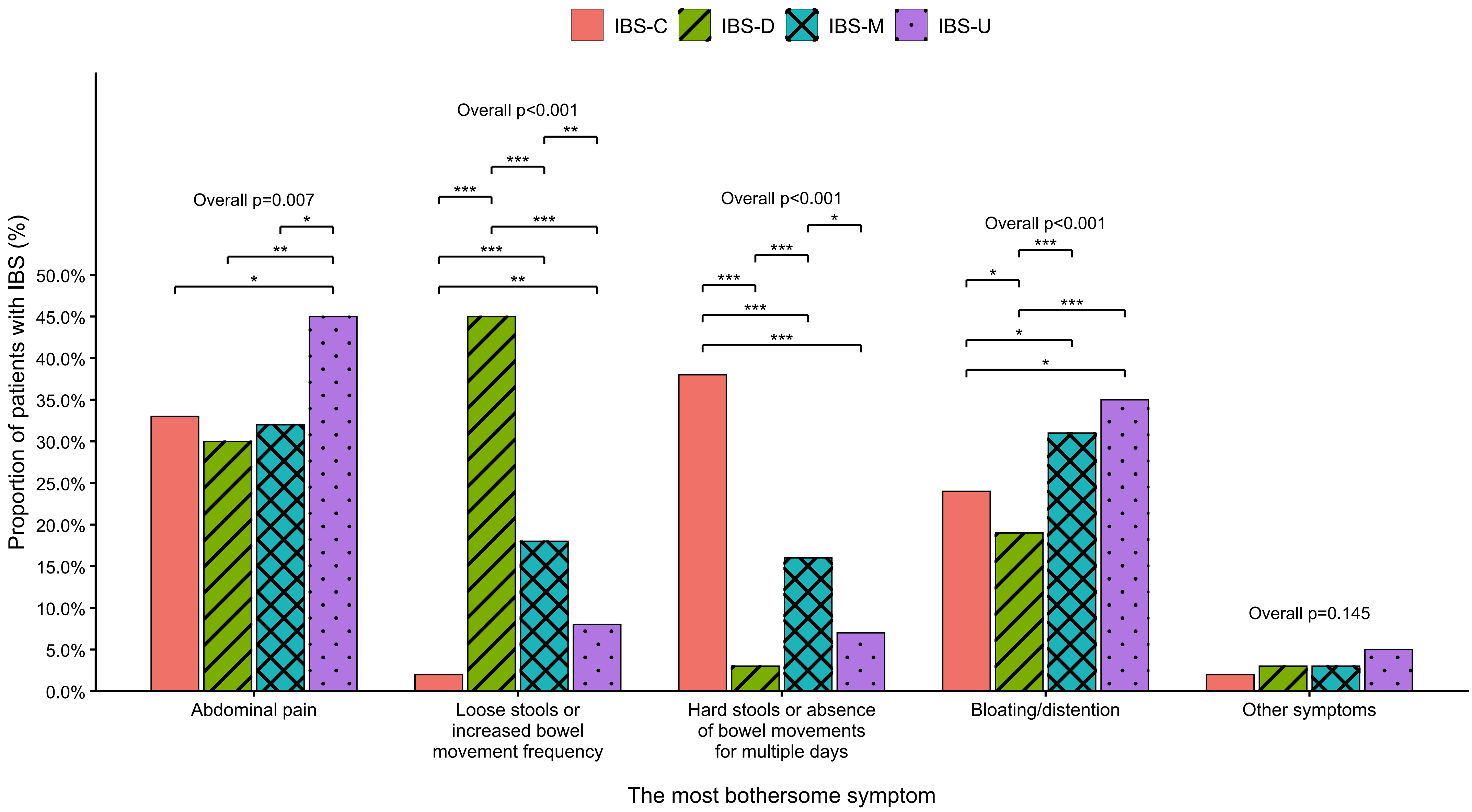

IBS-C
IBS-D
IBS-M
IBS-U
Proportion of patients with IBS (%)
50.0%
45.0%
40.0%
35.0%
30.0%
25.0%
20.0%
15.0%
10.0%
5.0%
0.0%
Overall p=0.007
Overall p<0.001
Overall p<0.001
Overall p<0.001
Overall p=0.145
Abdominal pain
Loose stools or increased bowel movement frequency
Hard stools or absence of bowel movements for multiple days
Bloating/distention
Other symptoms
The most bothersome symptom
