# Supplementary material for: Distinct pain profiles across irritable bowel syndrome subtypes: results from the Rome Foundation Global Epidemiology Study
Source: Intern Emerg Med. 2026 May 8;21(6):2065–75. doi: 10.1007/s11739-026-04375-1 (PMC13582041; doi:10.1007/s11739-026-04375-1)
Supplement: Supplementary file 2 [file 11739_2026_4375_MOESM2_ESM.pdf]

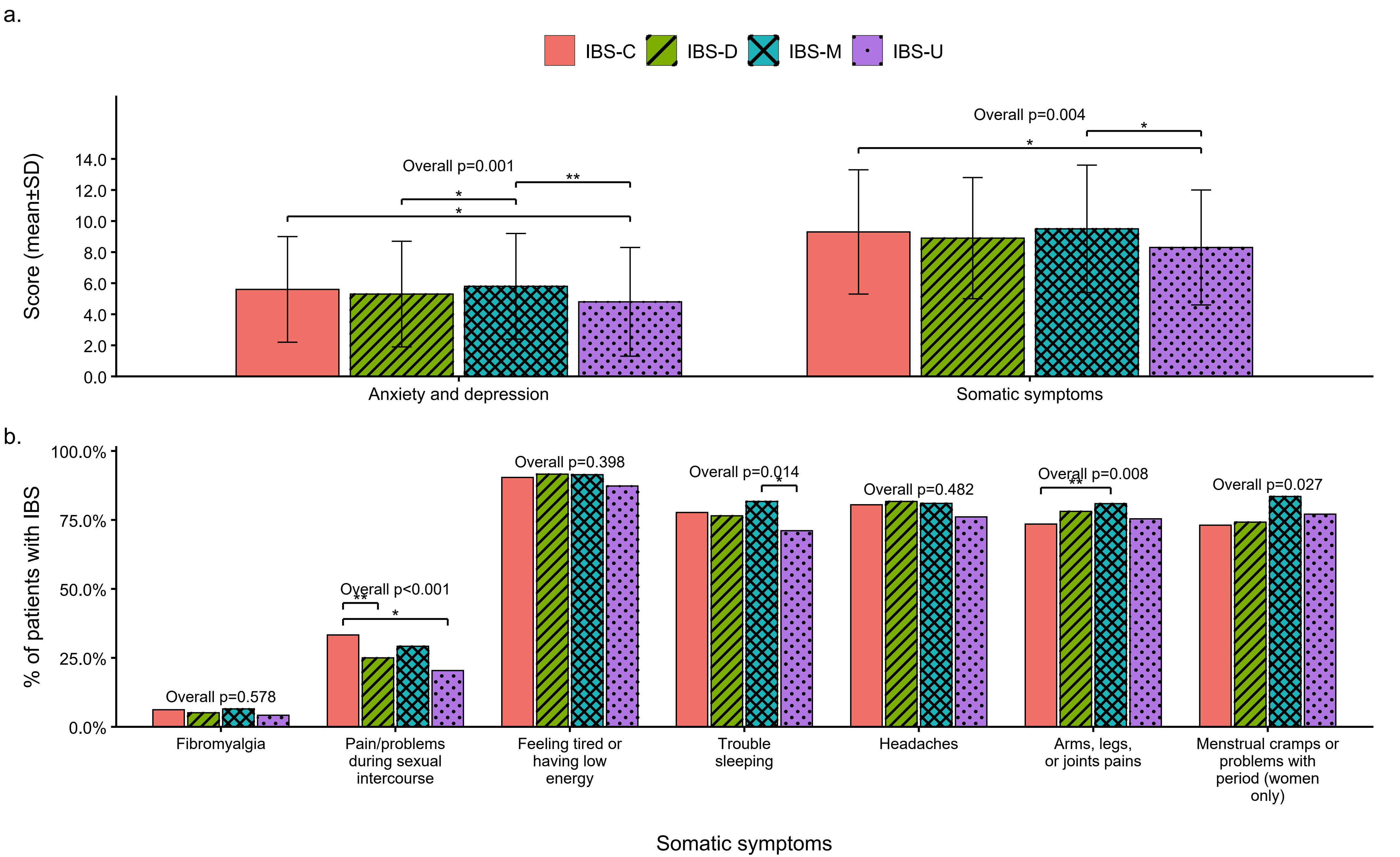

a.
IBS-C
IBS-D
IBS-M
IBS-U
Score (mean±SD)
Overall p=0.001
Overall p=0.004
Anxiety and depression
Somatic symptoms
b.
% of patients with IBS
Overall p=0.578
Overall p<0.001
Overall p=0.398
Overall p=0.014
Overall p=0.482
Overall p=0.008
Overall p=0.027
Fibromyalgia
Pain/problems during sexual intercourse
Feeling tired or having low energy
Trouble sleeping
Headaches
Arms, legs, or joints pains
Menstrual cramps or problems with period (women only)
Somatic symptoms
