# Supplementary material for: Distinct pain profiles across irritable bowel syndrome subtypes: results from the Rome Foundation Global Epidemiology Study
Source: Intern Emerg Med. 2026 May 8;21(6):2065–75. doi: 10.1007/s11739-026-04375-1 (PMC13582041; doi:10.1007/s11739-026-04375-1)
Supplement: Supplementary file 3 [file 11739_2026_4375_MOESM3_ESM.docx]

*Supplementary Table 1. Pairwise comparisons of baseline sociodemographic characteristics between IBS subtypes, adjusted for multiple testing using the Holm method.*

| *Variable* | *Comparison* | *Adjusted p-value (Holm)* |
| --- | --- | --- |
| Sex | IBS-C vs IBS-D | <0.001 |
| Sex | IBS-C vs IBS-M | 0.569 |
| Sex | IBS-C vs IBS-U | 0.111 |
| Sex | IBS-D vs IBS-M | 0.002 |
| Sex | IBS-D vs IBS-U | 0.680 |
| Sex | IBS-M vs IBS-U | 0.381 |
| BMI (kg/m2) | IBS-C vs IBS-D | 0.007 |
| BMI (kg/m2) | IBS-C vs IBS-M | 0.400 |
| BMI (kg/m2) | IBS-C vs IBS-U | 0.107 |
| BMI (kg/m2) | IBS-D vs IBS-M | 0.400 |
| BMI (kg/m2) | IBS-D vs IBS-U | 0.821 |
| BMI (kg/m2) | IBS-M vs IBS-U | 0.400 |
| BMI categories |  |  |
| Normal weight | IBS-C vs IBS-D | 0.001 |
| Normal weight | IBS-C vs IBS-M | 0.515 |
| Normal weight | IBS-C vs IBS-U | 0.125 |
| Normal weight | IBS-D vs IBS-M | 0.093 |
| Normal weight | IBS-D vs IBS-U | 1.000 |
| Normal weight | IBS-M vs IBS-U | 0.515 |
| Obesity | IBS-C vs IBS-D | 0.049 |
| Obesity | IBS-C vs IBS-M | 1.000 |
| Obesity | IBS-C vs IBS-U | 1.000 |
| Obesity | IBS-D vs IBS-M | 0.424 |
| Obesity | IBS-D vs IBS-U | 1.000 |
| Obesity | IBS-M vs IBS-U | 1.000 |
| Abbreviations: BMI, Body mass index, IBS-C, Constipation predominant irritable bowel syndrome, IBS-D, Diarrhea predominant irritable bowel syndrome, IBS-M, Irritable bowel syndrome mixed type, IBS-U, Unclassified irritable bowel syndrome  Pairwise comparisons are reported exclusively for variables that demonstrated a statistically significant overall difference across IBS subtypes (overall p < 0.05) | | |

*Supplementary Table 2. Frequency of consumption of food groups and food avoidance by IBS subtypes*

|  | *IBS-C (n=712)* | *IBS-D (n=629)* | *IBS-M (n=712)* | *IBS-U (n=142)* | *Overall p-value* |
| --- | --- | --- | --- | --- | --- |
| *Intake of food group (days/week)^c^, mean ± SD* |  |  |  |  |  |
| - Dairy products | 4.8±2.3^a^ | 4.3±2.5^b^ | 4.5±2.4^a,b^ | 4.3±2.4^a,b^ | 0.003 |
| - Meat | 4.1±2.1 | 4.2±2.1 | 4.1±2.0 | 3.9±2.1 | 0.405 |
| - Fish | 1.7±1.5 | 1.7±1.5 | 1.6±1.4 | 1.4±1.4 | 0.065 |
| - Eggs | 2.9±1.9 | 2.7±1.9 | 2.8±1.9 | 2.9±1.9 | 0.048 |
| - Vegetables and legumes | 4.9±1.9 | 4.7±2.0 | 4.9±2.0 | 4.5±2.1 | 0.018 |
| - Fruits | 4.3±2.1 | 4.1±2.3 | 4.3±2.3 | 4.0±2.3 | 0.160 |
| - Bread (including pizza, pita and tortillas) | 5.0±2.2 | 4.8±2.2 | 5.1±2.1 | 5.1±2.2 | 0.129 |
| - Pasta | 2.7±1.8^a^ | 2.4±1.8^b^ | 2.6±1.8^a,b^ | 2.3±1.7^a,b^ | 0.014 |
| - Rice | 3.1±2.2 | 3.0±2.2 | 3.0±2.3 | 3.2±2.2 | 0.760 |
| - Tofu | 0.7±1.5 | 0.7±1.3 | 0.7±1.4 | 0.5±1.3 | 0.180 |
| *Food avoidance^d^, % (n/N Italy)* | 53.3 (16/30) | 51.1 (23/45) | 40.0 (10/25) | 33.3 (1/3) | 0.705 |

Abbreviations: IBS-C, Constipation predominant irritable bowel syndrome; IBS-D, Diarrhea predominant irritable bowel syndrome; IBS-M, Irritable bowel syndrome mixed type; IBS-U, Unclassified irritable bowel syndrome; n, Number.

Pairwise comparisons were adjusted for multiple testing using the Holm method.

Groups sharing the same superscript letter (a, b) are not significantly different (p > 0.05)

^c^Dietary intake was assessed using the Food Frequency Questionnaire (FFQ)

^d^Food avoidance was reported exclusively by the Italian participants (n=2,063) in response to the question: "In your experience, have you eliminated particular foods from your diet that you believe are responsible for your gastrointestinal disorders? Percentages are therefore calculated using Italy-specific denominators (N Italy) for each IBS subtype.

*Supplementary Table 3. Holm-adjusted pairwise comparisons of dietary intake frequencies between IBS subtypes.*

| *Variable* | *Comparison* | *Adjusted p-value (Holm)* |
| --- | --- | --- |
| *Intake of food group (days/week)* |  |  |
| Dairy products | IBS-C vs IBS-D | 0.002 |
| Dairy products | IBS-C vs IBS-M | 0.087 |
| Dairy products | IBS-C vs IBS-U | 0.247 |
| Dairy products | IBS-D vs IBS-M | 0.556 |
| Dairy products | IBS-D vs IBS-U | 1.000 |
| Dairy products | IBS-M vs IBS-U | 1.000 |
| Eggs | IBS-C vs IBS-D | 0.082 |
| Eggs | IBS-C vs IBS-M | 0.697 |
| Eggs | IBS-C vs IBS-U | 0.697 |
| Eggs | IBS-D vs IBS-M | 0.592 |
| Eggs | IBS-D vs IBS-U | 0.182 |
| Eggs | IBS-M vs IBS-U | 0.697 |
| Vegetables and legumes | IBS-C vs IBS-D | 0.263 |
| Vegetables and legumes | IBS-C vs IBS-M | 0.685 |
| Vegetables and legumes | IBS-C vs IBS-U | 0.182 |
| Vegetables and legumes | IBS-D vs IBS-M | 0.086 |
| Vegetables and legumes | IBS-D vs IBS-U | 0.685 |
| Vegetables and legumes | IBS-M vs IBS-U | 0.086 |
| Pasta | IBS-C vs IBS-D | 0.050 |
| Pasta | IBS-C vs IBS-M | 0.607 |
| Pasta | IBS-C vs IBS-U | 0.081 |
| Pasta | IBS-D vs IBS-M | 0.264 |
| Pasta | IBS-D vs IBS-U | 0.607 |
| Pasta | IBS-M vs IBS-U | 0.249 |
| *Abbreviations: IBS-C, Constipation predominant irritable bowel syndrome; IBS-D, Diarrhea predominant irritable bowel syndrome; IBS-M, Irritable bowel syndrome mixed type; IBS-U, Unclassified irritable bowel syndrome.*  Pairwise comparisons are reported exclusively for variables that demonstrated a statistically significant overall difference across IBS subtypes (overall p < 0.05) | | |

*Supplementary Table 4. Comparison of clinical phenotypes between IBS subtypes (significant IBS-C versus IBS-D comparisons).*

|  | *IBS-C (n=712)* | *IBS-D (n=629)* | *IBS-M (n=712)* | *IBS-U (n=142)* | *Overall p-value* |
| --- | --- | --- | --- | --- | --- |
| Frequency of pain in the epigastric or right upper abdominal region^e^, median (IQR) | 4.0 (2.0-5.0)^a^ | 3.0 (2.0-5.0)^b^ | 4.0 (2.0-5.0)^a^ | 3.0 (1.0-4.3)^c^ | <0.001 |
| % Pain episodes that escalate to a sustained severe intensity, mean ± SD | 44.2±29.4^a^ | 38.1±29.3^b^ | 42.9±29.2^a^ | 29.4±27.4^c^ | <0.001 |
| % Pain episodes relieved by postural changes, median (IQR) | 50.0 (30.0-70.0)^a^ | 40.0 (10.0-60.0)^b^ | 40.0 (10.0-60.0)^b^ | 50.0 (20.0-70.0)^a,b^ | 0.004 |
| % Pain episodes accompanied by back pain or right shoulder blade pain, mean±SD | 36.7±32.2^a^ | 30.9±30.3^b^ | 35.4±32.9^a,b^ | 30.6±30.1^a,b^ | 0.007 |
| % Use of physician-prescribed pain medication at least once a week, % (n) | 39.3 (280)^a^ | 31.8 (200)^b^ | 36.7 (261)^a,b^ | 30.3 (43)^a,b^ | 0.016 |
| % Pain episodes with stool consistency changes, median (IQR) | 60.0 (50.0-80.0)^a^ | 70.0 (50.0-90.0)^b^ | 70.0 (50.0-80.0)^b^ | 50.0 (30.0-60.0)^c^ | <0.001 |
| % Pain episodes with altered stool frequency, mean±SD | 57.8±25.0^a^ | 61.3±24.5^b^ | 60.3±25.2^a,b^ | 43.9±24.2^c^ | <0.001 |
| IBS severity scale score, mean±SD | 256.4±100.3^a^ | 238.5±104.1^b^ | 259.0±102.9^a^ | 195.3±104.9^c^ | <0.001 |
| Dissatisfaction with bowel functioning, mean±SD | 65.3±26.2^a^ | 57.3±28.4^b^ | 62.4±27.6^a^ | 44.4±27.9^c^ | <0.001 |
| The most bothersome symptom, % (n) |  |  |  |  |  |
| - Abdominal pain | 33.1 (236)^a^ | 30.2 (190)^a^ | 31.5 (224)^a^ | 45.1 (64)^b^ | 0.007 |
| - Loose stools or increased bowel movement frequency | 2.4 (17)^a^ | 44.7 (281)^b^ | 18.0 (128)^c^ | 8.5 (12)^d^ | <0.001 |
| - Hard stools or absence of bowel movements | 38.5 (274)^a^ | 3.2 (20)^b^ | 16.2 (115)^c^ | 7.0 (10)^b^ | <0.001 |
| - Bloating/distention | 24.2 (172)^a^ | 18.9 (119)^b^ | 31.2 (222)^c^ | 34.5 (49)^c^ | <0.001 |
| - Other symptoms | 1.8 (13) | 3.0 (19) | 3.2 (23) | 4.9 (7) | 0.145 |

Abbreviations: IBS-C, Constipation predominant irritable bowel syndrome; IBS-D, Diarrhea predominant irritable bowel syndrome; IBS-M, Irritable bowel syndrome mixed type; IBS-U, Unclassified irritable bowel syndrome; IQR, Interquartile range; n, Number; SD, Standard deviation.

Pairwise comparisons were adjusted for multiple testing using the Holm method.

Superscript letters (a, b, c, d) indicate pairwise comparisons: groups sharing the same letter are not significantly different (adjusted p > 0.05).

^e^This item was scored by a scale of 0-8, with "0" meaning "never", and "8" meaning "multiple times per day or all day", and was assessed by the following question: "In the last 6 months how often did you have pain in the middle/right side of the upper abdomen?"

*Supplementary Table 5. Comparison of clinical phenotypes between IBS subtypes (non-significant IBS-C versus IBS-D comparisons)*

|  | *IBS-C (n=712)* | *IBS-D (n=629)* | *IBS-M (n=712)* | *IBS-U (n=142)* | *Overall p-value* |
| --- | --- | --- | --- | --- | --- |
| % Frequency of pain/burning in the epigastric region that interfered with daily activities^c^, mean±SD | 29.0±23.3^a,b^ | 25.9±21.5^a^ | 29.7±22.3^b^ | 24.7±21.8^a,b^ | 0.004 |
| % Epigastric pain episodes relieved by bowel movement or gas passage, median (IQR) | 60.0 (40.0-70.0) | 50.0 (30.0-70.0) | 60.0 (37.5-80.0) | 55.0 (30.0-70.0) | 0.215 |
| % Pain episodes lasting >30 minutes, mean±SD | 50.7±27.9^a^ | 47.0±29.2^a^ | 49.7±29.3^a^ | 36.3±29.1^b^ | <0.001 |
| % Pain episodes relieved by acid-suppressive therapy, median (IQR) | 40.0 (0.0-60.0) | 30.0 (0.0-60.0) | 30.0 (0.0-60.0) | 40.0 (0.0-62.5) | 0.078 |
| Abdominal pain frequency (days per 10-day period)^d^, median (IQR) | 5.0 (3.0-7.0) | 4.0 (3.0-6.0) | 5.0 (3.0-7.0) | 5.0 (3.0-6.0) | 0.174 |
| Extent to which gastrointestinal symptoms affect daily life, median (IQR)^e^ | 60.0 (40.0-70.0)^a^ | 50.0 (30.0-70.0)^a^ | 60.0 (30.0-80.0)^a^ | 40.0 (15.0-60.0)^b^ | <0.001 |
| Onset of pain or burning in the epigastric region at least six months ago, % (n) | 84.8 (451) | 84.4 (383) | 85.2 (472) | 76.0 (76) | 0.131 |
| % Abdominal pain episodes occurring near defecation, median (IQR)^f^ | 60.0 (40.0-80.0) | 60.0 (40.0-80.0) | 60.0 (40.0-80.0) | 50.0 (40.0-70.0) | 0.059 |
| % Pain episodes exacerbated by menstruation^g^, median (IQR) | 80.0 (40.0-100.0) | 80.0 (40.0-100.0) | 80.0 (50.0-100.0) | 70.0 (30.0-100.0) | 0.160 |
| % Pain episodes triggered or exacerbated by meals, median (IQR) | 50.0 (30.0-70.0)^a^ | 50.0 (30.0-70.0)^a^ | 50.0 (30.0-70.0)^a^ | 40.0 (20.0-60.0)^b^ | 0.002 |
| % Pain episodes that limit/restrict usual activities^h^, median (IQR) | 50.0 (30.0-70.0)^a^ | 50.0 (30.0-70.0)^a^ | 50.0 (30.0-70.0)^a^ | 35.0 (20.0-60.0)^b^ | <0.001 |
| Continuous or almost continuous abdomen pain^i^, % (n) | 49.4 (352) | 45.8 (288) | 47.5 (338) | 38.0 (54) | 0.081 |
| % Pain episodes with complete resolution between occurrences, median (IQR) | 50.0 (30.0-80.0) | 50.0 (30.0-80.0) | 50.0 (30.0-80.0) | 55.0 (30.0-82.5) | 0.428 |
| % Pain episodes leading to activity restriction or emergency consultation, median (IQR) | 30.0 (10.0-50.0)^a^ | 30.0 (10.0-50.0)^a^ | 30.0 (10.0-50.0)^a^ | 10.0 (0.0-50.0)^b^ | <0.001 |
| % Upper abdominal pain episodes temporally related to bowel movements^j^, median (IQR) | 40.0 (20.0-70.0)^a^ | 40.0 (20.0-70.0)^a^ | 40.0 (20.0-70.0)^a^ | 20.0 (10.0-50.0)^b^ | <0.001 |
| % Pain episodes accompanied by nausea or vomiting, median (IQR) | 20.0 (0.0-50.0)^a^ | 20.0 (0.0-40.0)^a,b^ | 20.0 (0.0-50.0)^a^ | 10.0 (0.0-40.0)^b^ | 0.005 |
| % Pain episodes causing nocturnal awakening, median (IQR) | 30.0 (10.0-50.0)^a^ | 20.0 (0.0-50.0)^a,b^ | 20.0 (10.0-50.0)^a^ | 10.0 (0.0-32.5)^b^ | 0.003 |
| Weekly frequency of non-prescription pain medication use, % (n) | 41.3 (294) | 38.6 (243) | 45.5 (324) | 33.8 (48) | 0.016 |

Abbreviations: IBS-C, Constipation predominant irritable bowel syndrome; IBS-D, Diarrhea predominant irritable bowel syndrome; IBS-M, Irritable bowel syndrome mixed type; IBS-U, Unclassified irritable bowel syndrome; IQR, Interquartile range; n, Number; SD, Standard deviation.

Pairwise comparisons were adjusted for multiple testing using the Holm method.

Superscript letters (a, b) indicate pairwise comparisons: groups sharing the same letter are not significantly different (adjusted p > 0.05).

^c^Evaluated using the question "In the last 3 months, how often did you have pain/burning in the middle part of upper abdomen, that was so severe that it interfered with the usual activities" and was indicated by a % ranged from 0% to 100%, with 0% meaning “Never” and 100% meaning “Always".

^d^For example, "4" means pain occurred 4 out of 10 days.

^e^Extent to which gastrointestinal symptoms affect daily life was assessed by the following question: "How much did abdominal pain/discomfort/altered bowel functioning affect/interfere with life in general in the past 10 days?" and was scored by a number from 0 to 100, with 0 meaning “Not at all” and 100 meaning “completely”.

^f^Just before, during, or soon after.

^g^For women only.

^h^For example: work, household activities, and social events.

^i^Continuous means that it never goes away during waking hours.

^j^Abdomen pain happens just before, during, or immediately following defecations.

*Supplementary Table 6. Holm-adjusted pairwise comparisons of clinical phenotypes across IBS subtypes (significant IBS-C vs. IBS-D comparisons only).*

| *Variable* | *Comparison* | *Adjusted p-value (Holm)* |
| --- | --- | --- |
| Frequency of pain in the epigastric or right upper abdominal region | IBS-C vs IBS-D | 0.025 |
| Frequency of pain in the epigastric or right upper abdominal region | IBS-C vs IBS-M | 0.885 |
| Frequency of pain in the epigastric or right upper abdominal region | IBS-C vs IBS-U | <0.001 |
| Frequency of pain in the epigastric or right upper abdominal region | IBS-D vs IBS-M | 0.025 |
| Frequency of pain in the epigastric or right upper abdominal region | IBS-D vs IBS-U | 0.006 |
| Frequency of pain in the epigastric or right upper abdominal region | IBS-M vs IBS-U | <0.001 |
| % Pain episodes that escalate to a sustained severe intensity | IBS-C vs IBS-D | 0.003 |
| % Pain episodes that escalate to a sustained severe intensity | IBS-C vs IBS-M | 0.434 |
| % Pain episodes that escalate to a sustained severe intensity | IBS-C vs IBS-U | <0.001 |
| % Pain episodes that escalate to a sustained severe intensity | IBS-D vs IBS-M | 0.015 |
| % Pain episodes that escalate to a sustained severe intensity | IBS-D vs IBS-U | 0.003 |
| % Pain episodes that escalate to a sustained severe intensity | IBS-M vs IBS-U | <0.001 |
| % Pain episodes relieved by postural changes | IBS-C vs IBS-D | 0.012 |
| % Pain episodes relieved by postural changes | IBS-C vs IBS-M | 0.018 |
| % Pain episodes relieved by postural changes | IBS-C vs IBS-U | 1.000 |
| % Pain episodes relieved by postural changes | IBS-D vs IBS-M | 1.000 |
| % Pain episodes relieved by postural changes | IBS-D vs IBS-U | 0.348 |
| % Pain episodes relieved by postural changes | IBS-M vs IBS-U | 0.348 |
| % Pain episodes accompanied by back pain or right shoulder blade pain | IBS-C vs IBS-D | 0.007 |
| % Pain episodes accompanied by back pain or right shoulder blade pain | IBS-C vs IBS-M | 0.663 |
| % Pain episodes accompanied by back pain or right shoulder blade pain | IBS-C vs IBS-U | 0.226 |
| % Pain episodes accompanied by back pain or right shoulder blade pain | IBS-D vs IBS-M | 0.125 |
| % Pain episodes accompanied by back pain or right shoulder blade pain | IBS-D vs IBS-U | 0.951 |
| % Pain episodes accompanied by back pain or right shoulder blade pain | IBS-M vs IBS-U | 0.539 |
| Use of physician-prescribed pain medication at least once a week | IBS-C vs IBS-D | 0.029 |
| Use of physician-prescribed pain medication at least once a week | IBS-C vs IBS-M | 0.651 |
| Use of physician-prescribed pain medication at least once a week | IBS-C vs IBS-U | 0.265 |
| Use of physician-prescribed pain medication at least once a week | IBS-D vs IBS-M | 0.280 |
| Use of physician-prescribed pain medication at least once a week | IBS-D vs IBS-U | 0.802 |
| Use of physician-prescribed pain medication at least once a week | IBS-M vs IBS-U | 0.528 |
| % Pain episodes with stool consistency changes | IBS-C vs IBS-D | <0.001 |
| % Pain episodes with stool consistency changes | IBS-C vs IBS-M | 0.015 |
| % Pain episodes with stool consistency changes | IBS-C vs IBS-U | <0.001 |
| % Pain episodes with stool consistency changes | IBS-D vs IBS-M | 0.254 |
| % Pain episodes with stool consistency changes | IBS-D vs IBS-U | <0.001 |
| % Pain episodes with stool consistency changes | IBS-M vs IBS-U | <0.001 |
| % Pain episodes with altered stool frequency | IBS-C vs IBS-D | 0.023 |
| % Pain episodes with altered stool frequency | IBS-C vs IBS-M | 0.104 |
| % Pain episodes with altered stool frequency | IBS-C vs IBS-U | <0.001 |
| % Pain episodes with altered stool frequency | IBS-D vs IBS-M | 0.452 |
| % Pain episodes with altered stool frequency | IBS-D vs IBS-U | <0.001 |
| % Pain episodes with altered stool frequency | IBS-M vs IBS-U | <0.001 |
| IBS severity scale score | IBS-C vs IBS-D | 0.005 |
| IBS severity scale score | IBS-C vs IBS-M | 0.628 |
| IBS severity scale score | IBS-C vs IBS-U | <0.001 |
| IBS severity scale score | IBS-D vs IBS-M | 0.002 |
| IBS severity scale score | IBS-D vs IBS-U | <0.001 |
| IBS severity scale score | IBS-M vs IBS-U | <0.001 |
| Dissatisfaction with bowel functioning | IBS-C vs IBS-D | <0.001 |
| Dissatisfaction with bowel functioning | IBS-C vs IBS-M | 0.071 |
| Dissatisfaction with bowel functioning | IBS-C vs IBS-U | <0.001 |
| Dissatisfaction with bowel functioning | IBS-D vs IBS-M | 0.003 |
| Dissatisfaction with bowel functioning | IBS-D vs IBS-U | <0.001 |
| Dissatisfaction with bowel functioning | IBS-M vs IBS-U | <0.001 |
| The most bothersome symptom |  |  |
| Abdominal pain | IBS-C vs IBS-D | 0.821 |
| Abdominal pain | IBS-C vs IBS-M | 1.000 |
| Abdominal pain | IBS-C vs IBS-U | 0.035 |
| Abdominal pain | IBS-D vs IBS-M | 1.000 |
| Abdominal pain | IBS-D vs IBS-U | 0.006 |
| Abdominal pain | IBS-M vs IBS-U | 0.012 |
| Loose stools or increased bowel movement frequency | IBS-C vs IBS-D | <0.001 |
| Loose stools or increased bowel movement frequency | IBS-C vs IBS-M | <0.001 |
| Loose stools or increased bowel movement frequency | IBS-C vs IBS-U | 0.001 |
| Loose stools or increased bowel movement frequency | IBS-D vs IBS-M | <0.001 |
| Loose stools or increased bowel movement frequency | IBS-D vs IBS-U | <0.001 |
| Loose stools or increased bowel movement frequency | IBS-M vs IBS-U | 0.007 |
| Hard stools or absence of bowel movements | IBS-C vs IBS-D | <0.001 |
| Hard stools or absence of bowel movements | IBS-C vs IBS-M | <0.001 |
| Hard stools or absence of bowel movements | IBS-C vs IBS-U | <0.001 |
| Hard stools or absence of bowel movements | IBS-D vs IBS-M | <0.001 |
| Hard stools or absence of bowel movements | IBS-D vs IBS-U | 0.056 |
| Hard stools or absence of bowel movements | IBS-M vs IBS-U | 0.015 |
| Bloating/distention | IBS-C vs IBS-D | 0.048 |
| Bloating/distention | IBS-C vs IBS-M | 0.015 |
| Bloating/distention | IBS-C vs IBS-U | 0.041 |
| Bloating/distention | IBS-D vs IBS-M | <0.001 |
| Bloating/distention | IBS-D vs IBS-U | <0.001 |
| Bloating/distention | IBS-M vs IBS-U | 0.497 |
| Other symptoms | IBS-C vs IBS-D | 0.843 |
| Other symptoms | IBS-C vs IBS-M | 0.643 |
| Other symptoms | IBS-C vs IBS-U | 0.322 |
| Other symptoms | IBS-D vs IBS-M | 1.000 |
| Other symptoms | IBS-D vs IBS-U | 1.000 |
| Other symptoms | IBS-M vs IBS-U | 1.000 |

Abbreviations: IBS-C, Constipation predominant irritable bowel syndrome; IBS-D, Diarrhea predominant irritable bowel syndrome; IBS-M, Irritable bowel syndrome mixed type; IBS-U, Unclassified irritable bowel syndrome.

Pairwise comparisons are reported exclusively for variables that demonstrated a statistically significant overall difference across IBS subtypes (overall p < 0.05)

Supplementary Table 7. Holm-adjusted pairwise comparisons of clinical phenotypes across IBS subtypes (non-significant IBS-C vs. IBS-D comparisons)

| *Variable* | *Comparison* | *Adjusted p-value (Holm)* |
| --- | --- | --- |
| Frequency of pain/burning in the epigastric region that interfered with daily activities | IBS-C vs IBS-D | 0.103 |
| Frequency of pain/burning in the epigastric region that interfered with daily activities | IBS-C vs IBS-M | 0.848 |
| Frequency of pain/burning in the epigastric region that interfered with daily activities | IBS-C vs IBS-U | 0.178 |
| Frequency of pain/burning in the epigastric region that interfered with daily activities | IBS-D vs IBS-M | 0.010 |
| Frequency of pain/burning in the epigastric region that interfered with daily activities | IBS-D vs IBS-U | 0.848 |
| Frequency of pain/burning in the epigastric region that interfered with daily activities | IBS-M vs IBS-U | 0.076 |
| % Pain episodes lasting >30 minutes | IBS-C vs IBS-D | 0.090 |
| % Pain episodes lasting >30 minutes | IBS-C vs IBS-M | 0.557 |
| % Pain episodes lasting >30 minutes | IBS-C vs IBS-U | <0.001 |
| % Pain episodes lasting >30 minutes | IBS-D vs IBS-M | 0.243 |
| % Pain episodes lasting >30 minutes | IBS-D vs IBS-U | 0.001 |
| % Pain episodes lasting >30 minutes | IBS-M vs IBS-U | <0.001 |
| Extent to which gastrointestinal symptoms affect daily life | IBS-C vs IBS-D | 0.331 |
| Extent to which gastrointestinal symptoms affect daily life | IBS-C vs IBS-M | 0.648 |
| Extent to which gastrointestinal symptoms affect daily life | IBS-C vs IBS-U | <0.001 |
| Extent to which gastrointestinal symptoms affect daily life | IBS-D vs IBS-M | 0.202 |
| Extent to which gastrointestinal symptoms affect daily life | IBS-D vs IBS-U | <0.001 |
| Extent to which gastrointestinal symptoms affect daily life | IBS-M vs IBS-U | <0.001 |
| % Pain episodes triggered or exacerbated by meals | IBS-C vs IBS-D | 1.000 |
| % Pain episodes triggered or exacerbated by meals | IBS-C vs IBS-M | 1.000 |
| % Pain episodes triggered or exacerbated by meals | IBS-C vs IBS-U | 0.001 |
| % Pain episodes triggered or exacerbated by meals | IBS-D vs IBS-M | 1.000 |
| % Pain episodes triggered or exacerbated by meals | IBS-D vs IBS-U | 0.002 |
| % Pain episodes triggered or exacerbated by meals | IBS-M vs IBS-U | 0.001 |
| % Pain episodes that limit/restrict usual activities | IBS-C vs IBS-D | 0.892 |
| % Pain episodes that limit/restrict usual activities | IBS-C vs IBS-M | 0.190 |
| % Pain episodes that limit/restrict usual activities | IBS-C vs IBS-U | <0.001 |
| % Pain episodes that limit/restrict usual activities | IBS-D vs IBS-M | 0.190 |
| % Pain episodes that limit/restrict usual activities | IBS-D vs IBS-U | 0.001 |
| % Pain episodes that limit/restrict usual activities | IBS-M vs IBS-U | <0.001 |
| % Pain episodes leading to activity restriction or emergency consultation | IBS-C vs IBS-D | 0.964 |
| % Pain episodes leading to activity restriction or emergency consultation | IBS-C vs IBS-M | 1.000 |
| % Pain episodes leading to activity restriction or emergency consultation | IBS-C vs IBS-U | <0.001 |
| % Pain episodes leading to activity restriction or emergency consultation | IBS-D vs IBS-M | 1.000 |
| % Pain episodes leading to activity restriction or emergency consultation | IBS-D vs IBS-U | 0.003 |
| % Pain episodes leading to activity restriction or emergency consultation | IBS-M vs IBS-U | <0.001 |
| % Upper abdominal pain episodes temporally related to bowel movements | IBS-C vs IBS-D | 1.000 |
| % Upper abdominal pain episodes temporally related to bowel movements | IBS-C vs IBS-M | 1.000 |
| % Upper abdominal pain episodes temporally related to bowel movements | IBS-C vs IBS-U | <0.001 |
| % Upper abdominal pain episodes temporally related to bowel movements | IBS-D vs IBS-M | 1.000 |
| % Upper abdominal pain episodes temporally related to bowel movements | IBS-D vs IBS-U | <0.001 |
| % Upper abdominal pain episodes temporally related to bowel movements | IBS-M vs IBS-U | <0.001 |
| % Pain episodes accompanied by nausea or vomiting | IBS-C vs IBS-D | 0.312 |
| % Pain episodes accompanied by nausea or vomiting | IBS-C vs IBS-M | 0.312 |
| % Pain episodes accompanied by nausea or vomiting | IBS-C vs IBS-U | 0.049 |
| % Pain episodes accompanied by nausea or vomiting | IBS-D vs IBS-M | 0.068 |
| % Pain episodes accompanied by nausea or vomiting | IBS-D vs IBS-U | 0.181 |
| % Pain episodes accompanied by nausea or vomiting | IBS-M vs IBS-U | 0.012 |
| % Pain episodes causing nocturnal awakening | IBS-C vs IBS-D | 0.178 |
| % Pain episodes causing nocturnal awakening | IBS-C vs IBS-M | 0.881 |
| % Pain episodes causing nocturnal awakening | IBS-C vs IBS-U | 0.006 |
| % Pain episodes causing nocturnal awakening | IBS-D vs IBS-M | 0.178 |
| % Pain episodes causing nocturnal awakening | IBS-D vs IBS-U | 0.098 |
| % Pain episodes causing nocturnal awakening | IBS-M vs IBS-U | 0.006 |
| Weekly frequency of non-prescription pain medication use | IBS-C vs IBS-D | 0.658 |
| Weekly frequency of non-prescription pain medication use | IBS-C vs IBS-M | 0.466 |
| Weekly frequency of non-prescription pain medication use | IBS-C vs IBS-U | 0.466 |
| Weekly frequency of non-prescription pain medication use | IBS-D vs IBS-M | 0.077 |
| Weekly frequency of non-prescription pain medication use | IBS-D vs IBS-U | 0.658 |
| Weekly frequency of non-prescription pain medication use | IBS-M vs IBS-U | 0.077 |

Abbreviations: IBS-C, Constipation predominant irritable bowel syndrome; IBS-D, Diarrhea predominant irritable bowel syndrome; IBS-M, Irritable bowel syndrome mixed type; IBS-U, Unclassified irritable bowel syndrome

Pairwise comparisons are reported exclusively for variables that demonstrated a statistically significant overall difference across IBS subtypes (overall p < 0.05)

.

*Supplementary Table 8. Associations between pain-related characteristics and IBS subtype (IBS-C vs. IBS-D): unadjusted and adjusted logistic regression models*

|  | *Model 1: Unadjusted models* | | *Model 2: Adjusted for sociodemographic factors, somatic symptom burden and nutritional variables* | |
| --- | --- | --- | --- | --- |
|  | Odds Ratio (95% CI) | p–value | Adjusted Odds Ratio (95% CI) | p–value |
| Frequency of pain in the epigastric or right upper abdominal region | 1.08 (1.03-1.14) | 0.004 | 1.07 (1.01-1.13) | 0.025 |
| % Pain episodes that escalate to a sustained severe intensity | 1.08 (1.04-1.12) | <0.001 | 1.07 (1.02-1.12) | 0.003 |
| % Pain episodes relieved by postural changes | 1.06 (1.02-1.10) | 0.004 | 1.04 (0.99-1.09) | 0.106 |
| % Pain episodes accompanied by back pain or right shoulder blade pain | 1.06 (1.02-1.10) | 0.002 | 1.05 (1.01-1.09) | 0.022 |
| % Pain episodes with stool consistency changes | 0.91 (0.87-0.96) | <0.001 | 0.89 (0.85-0.95) | <0.001 |
| % Pain episodes with altered stool frequency | 0.94 (0.90-0.98) | 0.010 | 0.93 (0.88-0.98) | 0.003 |
| Use of physician-prescribed pain medication at least once a week | 1.39 (1.11-1.74) | 0.004 | 1.23 (0.95-1.58) | 0.116 |

Abbreviations: CI, Confidence interval.

Model 1: Unadjusted models represent crude logistic regression analyses.
Model 2: Adjusted for sex (male/female), BMI (kg/m²), somatic symptom (PHQ–12 score), and nutritional intake of dairy products and pasta using the forward method.

Odds ratios are presented per unit increase for continuous variables and relative to absence of exposure for binary variables.

OR > 1 indicates higher likelihood of IBS-C; OR < 1 indicates higher likelihood of IBS-D.

*Supplementary Table 9. Comparisons of psychological distress, extra-intestinal comorbidity, and general assessments between IBS subtypes*

|  | *IBS-C (n=712)* | *IBS-D (n=629)* | *IBS-M (n=712)* | *IBS-U (n=142)* | *Overall p-value* |
| --- | --- | --- | --- | --- | --- |
| *Psychological variables* | | | | | |
| Presence of anxiety and depression^c^, % (n) | 77.9 (555) | 75.7 (476) | 77.1 (549) | 69.0 (98) | 0.131 |
| Anxiety and depression score^c^, mean±SD | 5.6±3.4^a,b^ | 5.3±3.4^a,c^ | 5.8±3.4^b^ | 4.8±3.5^c^ | 0.001 |
| *Somatic symptoms* | | | | | |
| Somatic symptoms scale score^d^, mean±SD | 9.3±4.0^a^ | 8.9±3.9^a,b^ | 9.5±4.1^a^ | 8.3±3.7^b^ | 0.004 |
| Fibromyalgia^e^, % (n) | 6.2 (44) | 5.1 (32) | 6.5 (46) | 4.2 (6) | 0.578 |
| Pain or problems during sexual intercourse^f^, % (n) | 33.3 (237)^a^ | 25.0 (157)^b^ | 29.2 (208)^a,b^ | 20.4 (29)^b^ | <0.001 |
| Feeling tired or having low energy^f^, % (n) | 90.4 (644) | 91.6 (576) | 91.4 (651) | 87.3 (124) | 0.398 |
| Trouble sleeping^f^, % (n) | 77.7 (553)^a,b^ | 76.5 (481)^a.b^ | 81.7 (582)^a^ | 71.1 (101)^b^ | 0.014 |
| Headaches^f^, % (n) | 80.5 (573) | 81.7 (514) | 81.0 (577) | 76.1 (108) | 0.482 |
| Arms, legs, or joints pains^f^, % (n) | 73.5 (523)^a^ | 78.1 (491)^a,b^ | 80.9 (576)^b^ | 75.4 (107)^a,b^ | 0.008 |
| Menstrual cramps or problems with period (women only) ^f^, % (n) | 73.1 (356) | 74.2 (262) | 83.5 (390) | 77.1 (64) | 0.027 |
| *General assessments* | | | | | |
| Concerns about bowel functioning^g^, % (n) | 90.7 (646)^a^ | 89.0 (560)^a^ | 91.6 (652)^a^ | 69.0 (98)^b^ | <0.001 |
| Embarrassment discussing bowel function^h^, % (n) | 60.5 (431)^a^ | 58.3 (367)^a,b^ | 61.4 (437)^a^ | 47.2 (67)^b^ | 0.014 |

Abbreviations: IBS-C, Constipation predominant irritable bowel syndrome; IBS-D, Diarrhea predominant irritable bowel syndrome; IBS-M, Irritable bowel syndrome mixed type; IBS-U, Unclassified irritable bowel syndrome; n, Number; SD, Standard deviation.

Pairwise comparisons were adjusted for multiple testing using the Holm method.

Superscript letters (a, b) indicate pairwise comparisons: groups sharing the same letter are not significantly different (adjusted p > 0.05).

^c^Presence of anxiety and depression was defined as mild, moderate or severe levels of these disorders, anxiety and depression were evaluated by the PHQ-4 questionnaire, while 0-2 score relates to non-psychological distress, 3-5 score = mild, 6-8 score = moderate, and 9-12 score = severe psychological distress.

^d^Somatic symptom scale score was evaluated by the PHQ-12 questionnaire, without the menstrual symptom item.

^e^Fibromyalgia was evaluated by the following question: "Have you ever been diagnosed by a doctor as having fibromyalgia?"

^f^Pain or problems during sexual intercourse, feeling tired or having low energy, trouble sleeping, headaches, arms, legs or joints pain, menstrual cramps or problems with period were evaluated by the following questions: In the past 4 weeks, how much have you been bothered by any of the following problems?" followed by the relevant symptoms, with menstrual cramps or problems with period being applied for women only.

^g^Concern about bowel functioning was assessed with the question: "Are you concerned about your bowel functioning?" Responses of "somewhat" and "very" were combined into a single positive category and compared against the negative response ("no"), converting the item from a categorical to a dichotomous scale.

^h^Embarrassment discussing bowel function was assessed with the question: "Are you embarrassed to discuss your bowel functioning with others?" Responses of "somewhat" and "very" were combined into a single positive category and compared against the negative response ("no"), converting the item from a categorical to a dichotomous scale.

*Supplementary Table 10. Holm-adjusted pairwise comparisons of psychological distress, extra-intestinal comorbidity, and general assessments across IBS subtypes*

| *Variable* | *Comparison* | *Adjusted p-value (Holm)* |
| --- | --- | --- |
| Anxiety and depression score | IBS-C vs IBS-D | 0.260 |
| Anxiety and depression score | IBS-C vs IBS-M | 0.260 |
| Anxiety and depression score | IBS-C vs IBS-U | 0.029 |
| Anxiety and depression score | IBS-D vs IBS-M | 0.029 |
| Anxiety and depression score | IBS-D vs IBS-U | 0.188 |
| Anxiety and depression score | IBS-M vs IBS-U | 0.003 |
| Somatic symptoms scale score | IBS-C vs IBS-D | 0.130 |
| Somatic symptoms scale score | IBS-C vs IBS-M | 0.734 |
| Somatic symptoms scale score | IBS-C vs IBS-U | 0.035 |
| Somatic symptoms scale score | IBS-D vs IBS-M | 0.069 |
| Somatic symptoms scale score | IBS-D vs IBS-U | 0.234 |
| Somatic symptoms scale score | IBS-M vs IBS-U | 0.018 |
| Pain or problems during sexual intercourse | IBS-C vs IBS-D | 0.006 |
| Pain or problems during sexual intercourse | IBS-C vs IBS-M | 0.276 |
| Pain or problems during sexual intercourse | IBS-C vs IBS-U | 0.017 |
| Pain or problems during sexual intercourse | IBS-D vs IBS-M | 0.276 |
| Pain or problems during sexual intercourse | IBS-D vs IBS-U | 0.302 |
| Pain or problems during sexual intercourse | IBS-M vs IBS-U | 0.168 |
| Trouble sleeping | IBS-C vs IBS-D | 0.648 |
| Trouble sleeping | IBS-C vs IBS-M | 0.260 |
| Trouble sleeping | IBS-C vs IBS-U | 0.348 |
| Trouble sleeping | IBS-D vs IBS-M | 0.105 |
| Trouble sleeping | IBS-D vs IBS-U | 0.438 |
| Trouble sleeping | IBS-M vs IBS-U | 0.033 |
| Arms, legs, or joints pains | IBS-C vs IBS-D | 0.290 |
| Arms, legs, or joints pains | IBS-C vs IBS-M | 0.006 |
| Arms, legs, or joints pains | IBS-C vs IBS-U | 1.000 |
| Arms, legs, or joints pains | IBS-D vs IBS-M | 0.669 |
| Arms, legs, or joints pains | IBS-D vs IBS-U | 1.000 |
| Arms, legs, or joints pains | IBS-M vs IBS-U | 0.654 |
| Menstrual cramps or problems with period (women only) | IBS-C vs IBS-D | 0.528 |
| Menstrual cramps or problems with period (women only) | IBS-C vs IBS-M | 0.383 |
| Menstrual cramps or problems with period (women only) | IBS-C vs IBS-U | 0.221 |
| Menstrual cramps or problems with period (women only) | IBS-D vs IBS-M | 0.178 |
| Menstrual cramps or problems with period (women only) | IBS-D vs IBS-U | 0.157 |
| Menstrual cramps or problems with period (women only) | IBS-M vs IBS-U | 0.509 |
| Concerns about bowel functioning | IBS-C vs IBS-D | 0.693 |
| Concerns about bowel functioning | IBS-C vs IBS-M | 0.693 |
| Concerns about bowel functioning | IBS-C vs IBS-U | <0.001 |
| Concerns about bowel functioning | IBS-D vs IBS-M | 0.414 |
| Concerns about bowel functioning | IBS-D vs IBS-U | <0.001 |
| Concerns about bowel functioning | IBS-M vs IBS-U | <0.001 |
| Embarrassment discussing bowel function | IBS-C vs IBS-D | 0.896 |
| Embarrassment discussing bowel function | IBS-C vs IBS-M | 0.896 |
| Embarrassment discussing bowel function | IBS-C vs IBS-U | 0.022 |
| Embarrassment discussing bowel function | IBS-D vs IBS-M | 0.848 |
| Embarrassment discussing bowel function | IBS-D vs IBS-U | 0.080 |
| Embarrassment discussing bowel function | IBS-M vs IBS-U | 0.014 |

Abbreviations: IBS-C, Constipation predominant irritable bowel syndrome; IBS-D, Diarrhea predominant irritable bowel syndrome; IBS-M, Irritable bowel syndrome mixed type; IBS-U, Unclassified irritable bowel syndrome

Pairwise comparisons are reported exclusively for variables that demonstrated a statistically significant overall difference across IBS subtypes (overall p < 0.05)
